# Remote Sensing of the Nano-Rheological Properties of Soft Materials Using Magnetic Nanoparticles and Magnetic AC Susceptometry

**DOI:** 10.3390/nano13010067

**Published:** 2022-12-23

**Authors:** Sobhan Sepehri, Johanna Andersson, Vincent Schaller, Cordula Grüttner, Mats Stading, Christer Johansson

**Affiliations:** 1 Digital Systems, RISE Research Institutes of Sweden, Arvid Hedvalls Backe 4, SE-41133 Gothenburg, Sweden; 2 Bioeconomy and Health, RISE Research Institutes of Sweden, Frans Perssons väg 6, SE-40229 Gothenburg, Sweden; 3 Micromod Partikeltechnologie GmbH, Friedrich-Barnewitz-Str. 4, D-18119 Rostock, Germany

**Keywords:** magnetic nanoparticles, AC susceptibility, viscoelastic properties, nano-rheology, soft materials

## Abstract

We have developed a nano-rheological characterization tool to extract the frequency- and scale-dependent rheological properties of soft materials during oral processing. Taking advantage of AC susceptometry, the dynamic magnetization of magnetic nanoparticles blended in the matrix material is measured. The magnetic AC susceptibility spectra of the particles are affected by the viscosity and mechanical modulus of the matrix material and provide the rheological properties of the matrix. Commercially available iron-oxide magnetic nanoparticles with 80 and 100 nm particle sizes are used as tracers in the frequency range of 1 Hz–10 kHz. The AC susceptibility is measured using two differentially connected coils, and the effects of the sample temperature and distance with respect to the detection coils are investigated. The developed measurement setup shows the feasibility of remote nano-rheological measurements up to 2 cm from the coil system, which can be used to, e.g., monitor the texture of matrix materials during oral processing.

## 1. Introduction

Studying the viscoelastic properties of a matrix material demands measurement equipment that consists of millimeter- to centimeter-sized mechanical objects that deform the matrix material at a set shear strain or stress, either at a small, non-destructive scale or at a larger scale, breaking or reforming the material. The method using large deformation in shear is usually referred to as viscometry, whereas small-amplitude harmonic deformation is called SAOS for Small-Amplitude Oscillatory Shear. Viscometry gives the true shear viscosity, and from the amplitude and phase analysis in SAOS, the dynamic shear viscosity and elastic moduli of the material are determined in the linear viscoelastic region [1]. However, these rheological measurements do not consider all of the dynamic transformations and complex aspects of oral processing, such as bolus rheology, palate pressure, biting force, etc. This causes inconsistencies between classical viscometry results and the sensory responses of subjects [2]. To overcome this issue, non-invasive methods are used to monitor the dynamic changes in food, and sensors are attached to subjects to track muscles and organs related to the oral processing of food. For instance, tongue pressure sensing using pressure transducers was used to observe palatal and swallowing pressures [3], biting pressures and forces were measured using sheet sensors [4], and ultrasound velocity profiling and X-ray video fluoroscopy were used for the in situ flow mapping of fluids with a focus on bolus rheology [5]. The complexity of the oral experience and the lack of characterization techniques for real time in vivo characterizations are the two major challenges in understanding food oral processing [6].

By mixing magnetic nanoparticles (MNPs) that undergo Brownian relaxation in the matrix material, it is possible to conduct the viscoelastic analysis in a non-invasive way [7,8,9,10,11]. In this case, the MNPs can be pictured as nanosized actuators and sensing objects. The viscoelastic properties can then be estimated by analyzing the frequency-dependent AC susceptibility of the MNPs [12,13,14]. Depending on the complexity of the matrix material, there are several viscoelastic models available for extracting the viscoelastic properties from AC susceptibility signals [9,11,15]. The goal of using MNPs is to remotely follow changes in rheological properties and thereby the texture and aggregation of food in oral processing. Iron-oxide MNPs are a suitable candidate particle system for this application. Iron-oxide-based MNPs are used in several biomedical applications, such as in diagnosis, actuation, imaging, and therapy [16,17]. Iron-oxide particles are also used as food colorants (E172).

In AC susceptibility measurements, an AC magnetic field is applied and subjects the MNPs to an oscillating magnetic torque. The real and imaginary components of the susceptibility of the MNPs are measured as a function of the applied field frequency. The MNPs respond to the applied field with a small rotation that is translated to a dynamic magnetic response characterized by the real and imaginary components of the AC susceptibility. The magnetic torque applied to the MNPs is about 10^12^ smaller than traditional rheologic measurements. However, it is applied at a much smaller length scale (about 5 orders of magnitude smaller).

Previously, we have reported on the different applications of AC susceptometry, for instance, magnetic characterization using the DynoMag system [18,19] and an AC susceptometer for biomedical sensing [20]. Here, we present an open-coil AC susceptometer system that enables AC susceptibility measurements a few millimeters outside the coil system. Therefore, it is possible to use AC susceptibility for the in vivo sensing of MNPs during food oral processing in the mouth.

## 2. Materials and Methods

Gelatin made from pig skin, Tørsleffs Favorit Gelatin (Haugen-Gruppen, Norrköping, Sweden), was used for gel experiments. The gels were prepared by stirring the gelatin in hot water (>80 °C) until dissolved. Commercially available bionized nanoferrite (BNF) iron-oxide (Fe_3_O_4_) MNPs (BNF-dextran, micromod Partikeltechnologie GmbH, Rostock, Germany) of 80 and 100 nm in size were added to the solution. The sample was then cooled down and stored at 8 °C. The prepared viscoelastic medium had 2 wt.% gelatin content and 5 mg/mL MNPs. Deionized (DI) water was used for all experiments. 

To use MNPs for rheological measurements in oral processing applications, the particles are required to have some key characteristics, such as a large magnetic moment, pH stability, and temperature stability. However, the most important feature is that the effective relaxation time of the MNP system is dominated by its Brownian relaxation. This means that the internal Néel relaxation time of the magnetic core is larger than the Brownian relaxation of the particle and the magnetic moment of the particle is locked in a particular direction during the stochastic rotational diffusion of the particle in the suspension solution [8,11,19].

The Brownian relaxation time/frequency in a Newtonian carrier liquid depends on the temperature *T*, the hydrodynamic volume of the particle *V_H_*, and the viscosity of the fluid *η* and is given by

(1) $$\tau_{B}=\frac{1}{2\pi \;f_{B}}=\frac{3\eta V_{H}}{k_{B}T}$$

where *k_B_* is the Boltzmann constant. From Equation (1), the viscosity of the Newtonian fluid can be extracted from the Brownian relaxation time of the suspended MNPs. A sensitive method to extract the relaxation time is the magnetic AC susceptibility technique. For a more complicated non-Newtonian fluid matrix, e.g., blood, there are theoretical approaches that use the complex AC susceptibility to extract frequency-dependent rheological data. Some examples are the generalized Debye model, which replaces the viscosity with complex viscosity in the dielectric Debye model [11], the Gemant–DiMarzio–Bishop (GDB) model known from dielectric spectroscopy relaxation [21,22], and the Raikher model [23]. The MNPs used in the experiments are multicore nanoparticles with median particle sizes of 80 and 100 nm, and they are thermally blocked and exhibit fully Brownian relaxation behavior. Therefore, they facilitate the investigation of the material properties of complex fluids at the nanometer scale in an optimal way.

**Figure 1:**
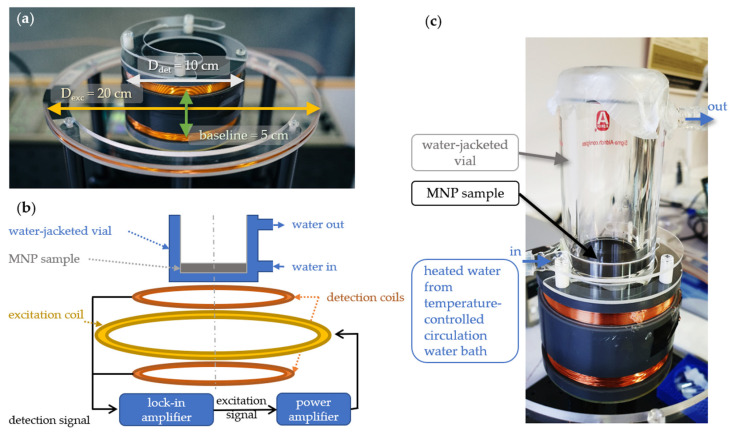
(**a**) A photograph of the excitation coil and detection coils used for magnetic AC susceptometry. (**b**) The schematic diagram shows the measurement setup and the connection of the coils to the lock-in amplifier. (**c**) A water-jacketed vial connected to a heated circulating water bath is used for changing the temperature of the MNP samples and is placed above the upper detection coil.

The measurement system comprises an excitation coil and two differentially connected pickup coils as detection coils. It is used for measuring the magnetic AC susceptibility of an MNP system in different fluid matrices. Both the excitation coil and the pickup coils are wound using enameled copper wire 0.4 mm in diameter. The pickup coils are centered coaxially with the excitation coil aligned in the middle of the two pickup coils in order to minimize the induced voltage in the detection coils. The two pickup coils form a basic first-order gradiometer, which is an ideal device for discriminating the signal from the surrounding environmental noise and the applied AC field. The baseline, the distance between the center of the two pickup coils, is around 5 cm, which is of the same order as the distance between the pickup coil and the test material in the mouth; see Figure 1a.

A lock-in amplifier (SR-830 Stanford instruments, Stanford Research Systems, Sunnyvale, CA, USA) is used to both generate the AC signal driving the excitation coil and measure the voltage drop in the detection coils (Figure 1b). In order to supply sufficiently high currents to the excitation coil, a power amplifier is placed before the coil to ensure a large excitation field amplitude. This creates a maximum field amplitude of 80 µT (at 10 Hz) at the center of the excitation coil. The in-phase and out-of-phase components of voltage induced in the detection coils due to the presence of the sample are measured by the lock-in amplifier. The two voltage components are proportional to the imaginary (out-of-phase) and real components (in-phase) of the frequency-dependent AC susceptibility, respectively.

To investigate the effect of temperature on the MNP fluid behavior, we have used a water-jacketed glass vial connected to a temperature-controlled heated circulating water bath. The sample is placed inside the jacketed glass vial located above one of the two detection coils (Figure 1c). Recirculating water between the jacketed glass vial and a temperature-controlled water bath allows us to regulate the samples’ temperature. The temperature of the samples can be set from ambient temperature up to 80 °C. Using a more versatile circulating water bath, it is possible to reach higher or lower temperatures. However, for the purposes of our studies, the implemented heated circulating bath is sufficient.

To compare the estimated viscoelastic properties with well-established methods, a rotational rheometer was used (ARES G2 rheometer; TA Instruments, New Castle, DE, USA) equipped with a 40 mm diameter parallel plate system maintained at 1 mm gap. The bottom plate of the equipment is temperature-controlled, and the measuring system is enclosed in a solvent-trap enclosure. SAOS is performed in the linear viscoelastic region giving the complex viscosity η*, storage modulus G′, and loss modulus G″ of the soft material.

## 3. Results and Discussion

### 3.1. Field Amplitude and Distance

**Figure 2:**
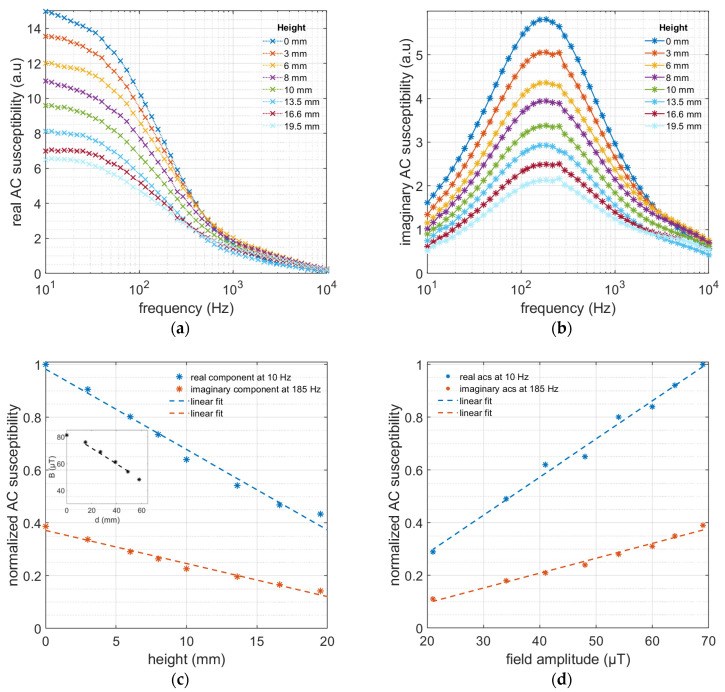
(**a**) The real and (**b**) imaginary components of magnetic AC susceptibility are plotted as a function of frequency at different distances with respect to the detection coil for the 100 nm MNP tracer. (**c**) Normalized real (at 10 Hz) and imaginary (at 185 Hz) components of the magnetic AC susceptibility of the 100 nm MNPs are plotted against the height measured from the detection coil. The lines are linear fits to the data. The inset shows the relation between the field amplitude and the distance from the center of the excitation coil and is measured using a Tesla meter. The line shows the linear dependence in the distance range where the MNP sample is placed. (**d**) Normalized real (at 10 Hz) and imaginary (at 185 Hz) components of magnetic AC susceptibility of the 100 nm MNPs versus the field amplitude scale linearly with the field amplitude in the range of 20–70 µT.

The performance of the sensor setup is determined by measuring the dynamic magnetic response of the MNPs at different distances relative to the pickup coil and field amplitudes. Figure 2a,b show the real and imaginary components of the AC susceptibility for the 100 nm MNPs at different distances with respect to the pickup coil. The amplitudes of both the real and imaginary components of the AC susceptibility signals decrease with increasing distance. This is due to both the decreased magnetic excitation field amplitude at the sample’s position as well as a lower magnetic coupling between the sample and the detection coil due to a longer distance between the two. Although the magnetization decreases with the decreasing magnetic field amplitude, the particles’ Brownian relaxation frequency is not affected. The Brownian relaxation time of the particles depends on the Langevin parameter *ζ* and is given by the following empirical formula [24]:

(2) $$\tau_{B}^{’}=\frac{\tau_{B}}{\sqrt{1+0.126\zeta^{1.72}}}$$

The Langevin parameter itself depends on the field amplitude and is given by

(3) $$\xi =\frac{mB}{k_{B}T}$$

where *m* is the magnetic moment of the particle, *B* is the applied magnetic field, *k_B_* is the Boltzmann constant, and *T* is the temperature. At small AC field amplitudes (smaller than 1 mT), this parameter is much smaller than 1, and the field amplitude does not affect Brownian relaxation. The relaxation time of the particles thus follows the widely known Brownian relaxation equation, Equation (1). Figure 2c demonstrates the linear dependence of normalized real components at the excitation frequency of 10 Hz and maximum imaginary components at 185 Hz vs. the distance to the detection coil. Both the real and imaginary components are normalized to the amplitude of the real component at 0 mm and 10 Hz. The decrease in the signal is due to the larger distance between the detection coil and the MNP sample, as previously discussed. In addition, the magnetic excitation field decreases with the increasing distance from the excitation coil, which lowers the sample’s magnetization. Using an F71 Teslameter (Lake Shore Cryotronics Inc., Westerville, OH, USA), the amplitude of the excitation field is measured at different distances from the center of the excitation coil. This dependency is not linear and inversely scales with the cube of the distance. However, in the distance range where the MNP sample is placed (20–40 mm from the center of the excitation coil), the field amplitude linearly depends on the distance. Figure 2d shows the linear dependency of the normalized AC susceptibility signal from 100 nm size MNPs to the magnetic field amplitude at zero distance with respect to the detection coil. Both the real and imaginary components are normalized to the amplitude of the real component at a frequency and field amplitude of 10 Hz and 70 µT, respectively. 

### 3.2. Temperature Dependence

During oral processing, the temperature of food changes in the mouth. Therefore, the temperature dependence of the Brownian relaxation of MNP tracers needs to be known. According to the Brownian relaxation time formula, increasing the temperature should result in a decrease (increase) in the relaxation time (frequency). However, the viscosity of the water as the suspension liquid is also affected by the temperature increase. The water viscosity decreases with the temperature increase and, in the range of 283 to 343 K (10 to 70 Celsius), follows an empirical formula [25]:

(4) $$\log\left(\frac{\eta_{T}}{\eta_{20}}\right)=\frac{A\left(293-T\right)-B{\left(T-293\right)}^{2}}{T+C}$$

where *T* is the temperature in Kelvin, *n_T_* is the water viscosity at temperature *T*, and *η*_20_ is the water viscosity at 20 Celsius (≈1.0020 mPa.s), with the constant parameters *A*, *B*, and *C* as *A* = 1.1709, *B* = 0.001827, and *C* = −183.07. There are more complicated and accurate formulations for the temperature-dependent viscosity of water; however, the above-mentioned formula is sufficiently correct for our purpose here. 

**Figure 3:**
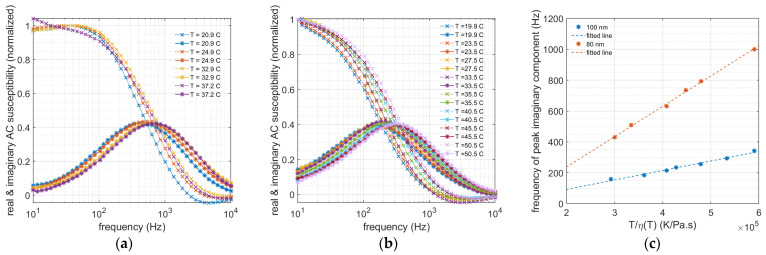
Normalized AC susceptibility of (**a**) 80 nm and (**b**) 100 nm MNP tracers versus magnetic excitation frequency plotted at different temperatures. (**c**) Frequency of peak imaginary component for both 80 nm and 100 nm MNPs at various temperatures plotted against T/η(T) shows a linear dependence.

Figure 3a,b show the AC susceptibility as a function of the excitation frequency at various temperatures for MNPs with median sizes of 80 and 100 nm. For both particle systems, the maximum imaginary components shift to higher frequencies as the temperature increases. Fitting the AC susceptibility data to the generalized Debye model with a log-normal size distribution, we can extract the particle size distribution at each temperature [19]. The particle size (volume-weighted) and the geometric standard deviation (width of the log-normal distribution) estimated from the Debye model are listed in Table 1. In the extraction of the particle size at each temperature, we have considered the change in the viscosity of the water from Equation (4). As can be seen from Table 1, the determined particle diameters are almost constant with the temperature, giving mean particle sizes of 97 nm and 140 nm for the 80 and 100 nm particle systems, respectively. These estimated diameters are higher than the ones reported by the supplier. This is simply because the generalized Debye model gives the volume-weighted values, while the supplier values of 80 and 100 nm are particle-number-weighted values. These results indicate that both MNP systems are quite stable in these temperature ranges and that there is no agglomeration or dissociation of particles. The determined particle sizes in Table 1 are in good agreement with earlier studies on these two MNP systems using other analysis methods [18,26].

The viscosity is a temperature-dependent parameter, which is present in the Brownian relaxation formula (Equation (1)). To reflect the effect of temperature on the viscosity, the Brownian relaxation frequency is plotted against T/η(T) (Figure 3c). Assuming that the hydrodynamic volume of the particles does not depend on the temperature, this dependence is linear. According to the Brownian relaxation frequency equation, Equation (1), the slope of this linear dependence is a constant value (=$\frac{k_{B}}{6\pi V_{H}}$) and inversely depends on the hydrodynamic volume of the particles (*V_H_*). The cubic of the ratio of the two particle diameters is then related to the slope of the frequency dependence in Figure 3c as follows:

(5) $${\left(\frac{d_{80\mathrm{nm}}}{d_{100\mathrm{nm}}}\right)}^{3}=\frac{V_{H}^{80\mathrm{nm}}}{V_{H}^{100\mathrm{nm}}}=\frac{Slope_{80\mathrm{nm}}}{Slope_{100\mathrm{nm}}}$$

Using the slopes of the lines of the best fit from Figure 3c (within the 98% confidence range), the above ratio is equal to 0.34±0.01. The estimated median diameter of the particles reported in Table 1, however, does not assume that the hydrodynamic volumes of the two particle systems are temperature independent. The cubic of the ratio of the two particle diameters estimated from the general Debye model is ${\left(\frac{80\;\mathrm{nm}}{100\;\mathrm{nm}}\right)}^{3}={\left(\frac{97\pm 2}{140\pm 2}\right)}^{3}=0.33\pm 0.03$. This ratio is remarkably close to the one estimated from the slopes of the lines of the best fit shown in Figure 3c. This shows that in these temperature ranges, one could assume that the particle size does not depend on the temperature. Therefore, these two MNPs are ideal for measuring the viscoelastic characteristics of the surrounding material matrix.

### 3.3. Gelatin Solution

**Figure 4:**
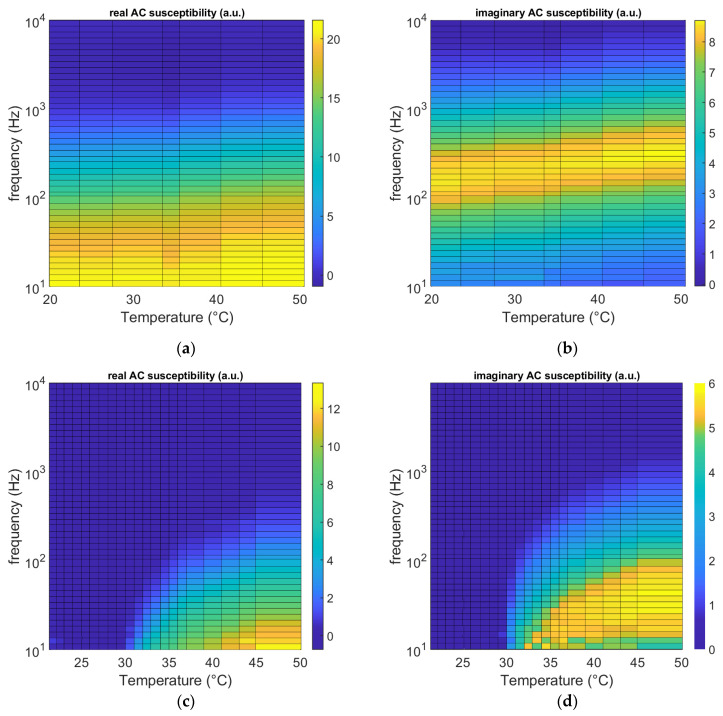
The real and imaginary components of AC susceptibility are plotted as a function of temperature and frequency for (**a**,**b**) MNPs suspended in DI water and (**c**,**d**) MNPs in the gelatin matrix. The color code represents the amplitude of the real and imaginary AC susceptibility signals.

To illustrate the capabilities of the measurement system, a viscoelastic model system consisting of a gelatin gel is used. To study the melting dynamics of the gelatin MNP gel, the sample is brought to 20 °C, and AC susceptibility is measured. The sample is then heated stepwise to 50 °C, where AC susceptibility is measured at each temperature step. The real and imaginary components of AC susceptibility are plotted against the temperature and frequency in contour plots for both MNPs in DI water and MNPs in a 2 wt.% gelatin solution in Figure 4. 

**Figure 5:**
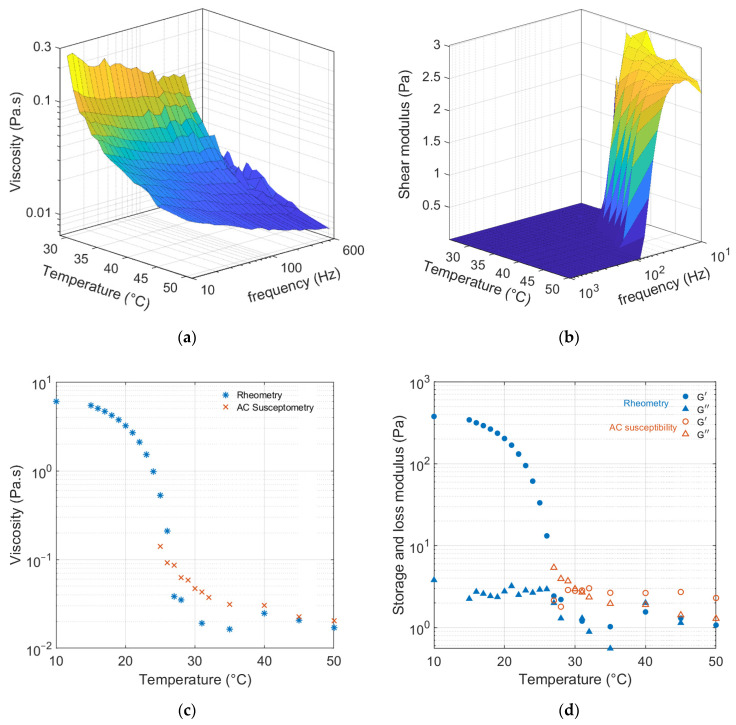
(**a**) Viscosity and (**b**) shear modulus of 100 nm MNPs in a matrix with 2 wt.% gelatin are estimated from AC susceptibility measurements. (**c**) Viscosity and (**d**) storage and loss moduli of the MNP–gelatin matrix at 10 Hz, plotted as a function of temperature measured by the rheometer (blue markers) and estimated from the AC susceptibility measurements (red markers). This trend shows the melting process of the gelatin matrix.

Figure 4a,b show the real and imaginary AC susceptibility of the 100 nm MNPs suspended in water. As the temperature increases, the maxima of the imaginary components shift to higher frequencies. This shift is due to both an increase in temperature and the lowered viscosity of the water at higher temperatures. The AC susceptibility spectrum for MNPs in the gelatin matrix (Figure 4c,d) is quite different from that for the MNPs suspended in water. Below 30 °C, the amplitudes of both the real and imaginary components of the AC susceptibility are zero in our frequency window. This is due to the almost total immobilization of the MNPs in the gelatin matrix, which brings the relaxation frequencies to very low frequencies that are outside our frequency window. At higher temperatures, the AC spectra start to appear with the peaks at low frequencies. Increasing the temperature further shifts the maximum position of the imaginary part to higher frequencies. To obtain the viscosity and shear modulus of the gelatin matrix, the Raiker model [9,23] is used. This model considers the matrix as a viscoelastic system with viscous and elastic terms in parallel. The real and imaginary components of viscosity are determined at each temperature and frequency. The shear modulus is then found by multiplying the angular frequency (ω=2πf) with the imaginary viscosity. The temperature and frequency dependence of the viscosity and shear modulus of the MNP–gelatin matrix are plotted in Figure 5a,b. These parameters can only be calculated if there is a real and imaginary susceptibility signal available from the MNP tracers. Therefore, the estimated values for these properties are only shown in the temperature and frequency domains where there is an AC susceptibility signal available. The viscosity of the MNP–gelatin matrix decreases with increasing temperature, which results in a faster Brownian relaxation time. 

These estimated values are quite close to what is measured for the same matrix system using conventional SAOS using a rheometer. A comparison of the rheometer shear measurements of the MNP–gelatin matrix with the AC susceptibility measurements is plotted in Figure 5c,d. These plots show the melting process of the matrix at 10 Hz for both methods. The estimated values from the AC susceptibility technique are quite close to the ones from conventional rheometer measurements. The difference in the viscosity and shear modulus of these two methods could be due to the torque applied to the gelatin matrix. The applied torque during the magnetic excitation of the MNPs in the AC susceptibility method is much smaller compared to the rheometer. 

## 4. Conclusions

The nanoscale rheology of complex fluids is studied using the frequency-dependent response of MNP tracers in an oscillating magnetic field. The method described here facilitates non-invasive nano-rheological measurements up to 2 cm from the coil system. Using a temperature-controlled water bath and a water-jacketed flask made it possible to measure the AC susceptibility of MNPs in both water and a gelatin matrix at different temperatures. Gelatin was chosen as a viscoelastic material, and its viscoelastic properties were characterized by adding 100 nm MNP tracers and measuring the MNPs’ AC susceptibility in the gelatin matrix. The method can be used for the remote monitoring of complex fluids and will be applied to measure the oral processing of matrix materials.

## Figures and Tables

**Table 1:** Hydrodynamic particle diameters (volume-weighted) and their geometrical standard deviations at each temperature for both 80 and 100 nm nanoparticles extracted from fitting the AC susceptibility measurement data with the generalized Debye model. The effect of temperature on the viscosity of water is estimated from the empirical formula given by Equation (4). The 80 nm and 100 nm sizes refer to number-weighted hydrodynamic particle diameters.

MNP System	100 nm							80 nm			
Temperature (°C)	20.1	23.5	27.5	33.5	35.5	40.5	45.5	20.9	24.9	32.9	37.2
Particle median diameter (nm)	144	142	140	139	141	139	137	97	95	97	100
Geometrical standard deviation (nm)	48	49	46	44	45	47	45	24	28	30	31

## Data Availability

Not applicable.
